# Management of Hypotension and Bradycardia Caused By Spinal Cord Injury. The Usefulness of Midodrine and Methylxanthines

**DOI:** 10.22037/ijpr.2019.1100824

**Published:** 2019

**Authors:** Mojtaba Mojtahedzadeh, Hamidreza Taghvaye-masoumi, Atabak Najafi, Mehrnoush Dianatkhah, Hamidreza Sharifnia, Maryam Shahrokhi

**Affiliations:** a *Department of Clinical Pharmacy, Faculty of Pharmacy, Tehran University of Medical Sciences, Tehran, Iran. *; b *Department of Anesthesiology and Intensive Care, School of Medicine, Tehran University of Medical Sciences, Tehran, Iran. *; c *Department of Clinical Pharmacy, Faculty of Pharmacy, Gilan University of Medical Sciences, Rasht, Iran.*

**Keywords:** Case report, Spinal cord injury, Midodrine, Methylxanthines, Hypotension, Bradycardia

## Abstract

Spinal cord injury is a devastating chronic condition resulting in temporary or permanent motor, sensory or autonomic dysfunction of the cord. The manifestation of spinal cord injury based on the severity and involved areas could be different. Numerous studies have demonstrated that bradycardia, hypotension, and orthostatic hypotension are present insignificant number of patients after spinal cord injury which peaks at 4^th^ day of injury. Although vasopressors are common drugs that have been used to restore blood pressure and heart rate in patients with neurogenic shock, there is limited data regarding pharmacologic management of bradycardia and hypotension after spinal cord injury. Midodrine is a potent vasopressor approved for the management of symptomatic orthostatic hypotension. Theophylline and aminophylline are methylxanthine derivatives. There are very few case reports concerning the use of midodrine and methylxanthines for treatment of hypotension in patients with spinal cord injury. In this case report and review of the articles we report a 45 year old woman with a diagnosis of spinal cord injury who was successfully managed with midodrine and aminophylline and then we review current case reports. Based on our case report and other available data, midodrine as well as methylxanthines can be suggested as therapeutic options for managing symptoms in spinal cord injury patients.

## Introduction

Spinal cord injury is a devastating chronic condition resulting in temporary or permanent motor, sensory or autonomic dysfunction of the cord. Motor accidents, falls, interpersonal violence, and sports are the most common causes of spinal cord injury. Direct trauma, compression of the vertebrae and ischemia due to damage on the spinal arteries can lead to sustained spinal cord injury. In 2017 there were approximately 285,000 alive persons with history of spinal cord injury in the United States (1). The manifestation of spinal cord injury based on the severity and involved areas could be different. It has been shown that injuries above C5 have the most association with cardiovascular abnormalities. Numerous studies have demonstrated that bradycardia, hypotension, and orthostatic hypotension are present in a significant number of patients after spinal cord injury which is mostly seen at 4^th^ day of injury (2, 3). 

Based on recently published guidelines, it is suggested that early surgery be considered as a treatment option in adult patients with spinal cord injury.

Although it is suggested that a 24-hour infusion of high-dose methylprednisolone sodium succinate has beneficial effect on adult patients with acute SCI, the guideline does not suggest 24-hour infusion of high-dose methylprednisolone to the adult patients present after 8 h with acute SCI (4).

Neurogenic shock is one of the most important complications of the spinal cord injury resulting from autonomic dysfunction and disturbance in the sympathetic outflow to the cardiovascular system and subsequent decreased cardiac output (CO), and systemic vascular resistance. Severe autonomic dysfunction may lead to hypotension, bradycardia, respiratory rate dysregulation, hypothermia, and peripheral vasodilation in injuries involvingT6 or higher. Early diagnosis and treatment of acute signs and symptoms are critical for successful management of the patients with neurogenic shock. Vasopressors are common drugs that have been used for years to restore blood pressure (target MAP of 85-90 mmHg for the first seven days) and heart rate (target HR of 60-100 beats per minute) in the patients with neurogenic shock. However, cardiac abnormalities after spinal cord injury are usually temporary and resolve after 6 to 8 weeks (5).

Treatment of cardiovascular complications consists of maintenance of euvolemia and substitution of alpha sympathetic agonists such as phenylephrine, ephedrine, dopamine, etc.

Midodrine is a potent vasopressor approved for the management of symptomatic orthostatic hypotension. Midodrine is a pro-drug and after administration rapidly is converted to the active metabolite (desglymidodrine) which is a selective alpha1-agonist and produces an increase in vascular resistance and elevation of blood pressure. There are very few case reports about the usage of midodrine for treatment of hypotension in the patients with spinal cord injury.

Theophylline and aminophylline are methylxanthine derivatives with two distinct mechanisms of action including inhibition of phosphodiesterase III and adenosine receptor antagonism. There are limited clinical experiences about use of methylxanthinesin the management of bradycardia secondary to spinal cord injury.

Here we have reported a patient with bradycardia and hypotension due to spinal cord injury who was successfully managed with theophylline and midodrine.

## Case report

A 45-year-old otherwise healthy woman with a diagnosis of spinal cord injury due to falling down the stairs about 13 h ago was admitted to our hospital. She wasn’t on any medication. Based on CT-scan findings her injuries included C5-6 dislocation causing severe cord contusion and compression with the manifestation of motor and sensory loss. The patient underwent neurosurgical intervention in order to fix the cervical dislocation and prevent vertebral compression. After the operation she was admitted to our ICU with BP = 99/45 mmHg and o2 saturation = 100%. On physical exam she has no active bleeding, clear respiratory sounds without distress and her force of left upper limb was 3/5. Her lab data and hemodynamic parameters were presented in Table 1. On day 1 of injury the patient developed hypotension (BP = 99/45 mm-Hg) and bradycardia (HR = 50 bpm). We maintained euvolemia based on frequent bedside echocardiography. According to our hospital protocol continuous infusion of methylprednisolone 100 mg/24 h was administered on day 1. Despite adequate hydration she still had bradycardia and hypotension, so dopamine was initiated to optimize blood pressure and heart rate. On day 2, because of continuing bradycardia, aminophylline infusion (10 mg/h) was administered.

On day-3after the injury, the patient was successfully weaned off dopamine, and midodrine was started (2.5 mg BD) and the dose was titrated to 5 mg three times a day. And 5 days later, aminophylline drip was discontinued because the patient’s HR was stable on 76 bpm without aminophylline. The patient did not have any further episodes of bradycardia or hypotension, so there was no need to start theophylline instead of aminophylline. Her BP was about 130/70 on midodrine, so we did not discontinue midodrine. No serious adverse effects including arrhythmia or central nervous system side effects were observed during therapy. She also received citalopram for improving her depression, melatonin for insomnia, ipratropium bromide and salbutamol and N-acetylcysteine, pregabalin for neuropathic pain, pantoprazole and prophylactic dose of enoxaparin.

Subsequently the patient was transferred to the neurosurgery ward and after one week, she was successfully discharged from the hospital on midodrine without any bradycardia or hypotension while she was paraplegic. She was advised to increase her salt and water intake and was appointed for our clinic to follow up on her BP, HR, and possibly tapering down of midodrine.

## Literature review

*Method*

Medline, Scopus, and Cochrane Database of Systematic Reviews were searched using these keywords: “methylxantine,” midodrine,” “aminophylline,” “theophylline,” spinal cord injury,” and “treatment”. All published articles from 1980 to 2017 were included in the search.

## Results

A total of 13 relevant human studies were found after excluding irrelevant articles (basic experimental studies, non-English language reports and studies that did not include clinical end-point assessments).

## Discussion

Bradycardia and hypotension have been observed in a large number of patients after spinal cord injury specially injuries involving the level of C5 or above (2). In this article we reported a patient with bradycardia and hypotension due to cervical spinal cord injury which was successfully treated with midodrine and aminophylline. 

There is limited data regarding the use of methylxanthines for the management of cervical spine injury related bradycardia. In 2005, Schulz-Stübner reported three patients with bradycardia due to spinal cord injury that were successfully treated with methylxanthines (intravenous aminophylline or oral theophylline). In two of the patients theophylline was used as a second line therapy after administration of anti-cholinergic agents (atropine and glycopyrolates) and the third patient received methylxanthine as a first line agent. Additionally, Theophylline therapy was associated with increased respiratory drive and minute ventilation in the treated patients. No serious adverse effects regarding methylxanthine administration were reported and the theophylline serum concentrations in all of the patients were below 3.4 mg/L (one case didn’t have theophylline plasma levels) (6). In 2004, Pasnoori reported two patients with acute cervical spinal cord injury who had bradycardia resistant to atropine. They successfully managed these patients using intravenous aminophylline (5). In 2007, Sakamoto described that sequential use of aminophylline and theophylline was effective and safe for the management of spinal cord injury induced bradycardia in one Japanese man whose bradycardia was refractory to atropine (7). In 2008, Whitman* et al. *described a patient with recurrent symptomatic bradycardia secondary to high cervical spinal cord injury who was treated successfully with administration of intravenous aminophylline. 

The theophylline plasma levels were 1.9-3.4 mg/L (8). In 2007, Weant* et al.* showed the effectiveness and safety of oral theophylline for the treatment of cervical spine injury induced symptomatic bradycardia and asystole in one patient. The patient′s serum theophylline concentrations were ≤ 3.2 mg/L (9). In 2010, Sadaka* et al.* published one case series including 6 cervical spine cord injury cases with bradycardia who were successfully managed with the administration of oral theophylline (via nasogastric tube). Oral theophylline was effective in all patients and no serious adverse effects were observed. Theophylline was used in four patients as a second line or adjunct therapy and in two patients as first line therapy. The theophylline plasma levels throughout the therapy in all patients were ≤7.6 mg/L (below the toxic range) (2).

There is limited data on the use of midodrine for management of hypotension secondary to spine injury. Midodrine as a selective oral alpha1-receptor agonist has been used for the treatment of spinal cord injury induced orthostatic hypotension in a few cases.

Nieshoff* et al.* in one double-blind, placebo-controlled, randomized trial on four patients suffering from cervical spine injury demonstrated that midodrine 10 mg orally was effective and safe for the management of orthostatic hypotension in this population (10). In 1991, Senard* et al.* reported that midodrine 10 mg orally has been beneficial for the treatment of orthostatic hypotension due to spine injury in one patient (11). In 2000, Barber* et al.* also described two patients with spinal cord injury related orthostatic hypotension that were successfully treated with midodrine (12). In 2001, Mukand* et al.* showed that midodrine was effective and safe for maintaining blood pressure and reducing orthostatic symptoms in one patient with cervical spine injury induced orthostatic hypotension (13). In a prospective dose-response trial on ten patients with chronic cervical spine injury in 2010, Wecht* et al. *suggested that midodrine 10 mg could be considered for the treatment of hypotension and orthostatic hypotension in this population (14). In 2014, Phillips* et al. *demonstrated that midodrine may improve orthostatic hypotension and cerebral blood flow velocity of the posterior cerebral artery in ten patients with history of spinal cord injury (15).

In our case we managed post cervical spine injury associated bradycardiaand hypotension in a fluid unresponsive patient with aminophylline and midodrine. Dopamine drip was changed to oral midodrine after 3 days of dopamine infusion. We did not measure aminophylline serum concentration; on the other hand we did not observe any adverse effect related to aminophylline or midodrine and the patient was successfully treated. In all above mentioned studies theophylline serum concentrations were below lower limit of therapeutic range (therapeutic range: 10-20 mg/L) and not only all the patients were successfully managed, but also none of them suffered from any serious adverse effects. Therefore, low doses of methylxanthines and midodrine have been shown to be safe and effective in post spine injuries. 

The patients with acute spinal injury may have disturbances in the absorption of medication from gastrointestinal tract that may affect pharmacokinetic and plasma concentration of the drugs. However, it has been shown that oral administration of theophylline was effective in case reports which were published by Schulz-Stübner, Sakamoto, Weant* et al*. and Sadaka* et al. *Midodrine also has been used orally in this population in some cases and its efficacy in elevation of blood pressure was 

demonstrated.

**Table 1:** Lab data and hemodynamic parameters

	**Day 1 Admission to the ICU**	**Day 3** **On Aminophylline and dopamine**	**Day4** **Off dopamine On Aminophylline** **and midodrine**	**Day 5** **On midodrine**	**Day 35, Discharge to the ward** **On midodrine**
HgB	9.9	9.6	9.7	9.5	10.1
Na	130	137	140	141	140
K	4.3	3.5	4	4.2	4.1
BUN	15	17	17	15	19
SrCr	0.69	0.8	0.8	0.9	0.7
BP	99/45	126/72	120/70	118/65	137/73
HR	50	76	70	65	73
RR	14	16	15	15	16

HgB: Hemoglobin; Na: Sodium; K: Potassium; BUN: Blood Urea Nitrogen; SrCr: Serum Creatinine; BP: Blood Pressure; HR: Heart Rate; RR: Respiratory Rate.

## Conclusion

Based on our case report and other evidences, midodrine as well as methylxanthines can be suggested as a therapeutic option for managing symptoms in the spinal cord injury patients. Major privilege for midodrine is the potential for oral administration. It does not need therapeutic drug monitoring and also has lower risk of arrhythmia. In addition, less drug interaction and more specific effect on BP are other positive points. Considering these privileges, midodrine would be a more suitable option for long-term therapy to maintain the blood pressure.

Further research is required to determine the optimal dosage and duration of methylxanthines and midodrine for this indication. In our patients, the decision to continue midodrine and discontinue aminophylline was made purely based on the clinical judgment of the overall recovery profile.

## Ethics approval and consent to participate

The patients were aware of the benefits and risks of these interventions and agreed to the therapy and data publication.
